# Nanopharmaceutics: Part II—Production Scales and Clinically Compliant Production Methods

**DOI:** 10.3390/nano10030455

**Published:** 2020-03-04

**Authors:** Eliana B. Souto, Gabriela F. Silva, João Dias-Ferreira, Aleksandra Zielinska, Fátima Ventura, Alessandra Durazzo, Massimo Lucarini, Ettore Novellino, Antonello Santini

**Affiliations:** 1 Department of Pharmaceutical Technology, Faculty of Pharmacy, University of Coimbra, Pólo das Ciências da Saúde, Azinhaga de Santa Comba, 3000-548 Coimbra, Portugal; gabriela.mgfs@gmail.com (G.F.S.); j.dias.ferreira@outlook.pt (J.D.-F.); zielinska-aleksandra@wp.pl (A.Z.); 2 CEB-Centre of Biological Engineering, University of Minho, Campus de Gualtar, 4710-057 Braga, Portugal; 3 Department of Biochemistry and Human Biology, Faculty of Pharmacy of University of Lisbon, 1649-003 Lisbon, Portugal; fatima.ventura@infarmed.pt; 4 CREA-Research Centre for Food and Nutrition, Via Ardeatina 546, 00178 Rome, Italy; alessandra.durazzo@crea.gov.it (A.D.); massimo.lucarini@crea.gov.it (M.L.); 5 Department of Pharmacy, University of Napoli Federico II, Via D. Montesano 49, 80131 Napoli, Italy; ettore.novellino@unina.it

**Keywords:** nanopharmaceutics, nanonutraceutics, legislation, lipid-based, polymer-based, metal-based, clinical requirements

## Abstract

Due the implementation of nanotechnologies in the pharmaceutical industry over the last few decades, new type of cutting-edge formulations—nanopharmaceutics—have been proposed. These comprise pharmaceutical products at the nanoscale, developed from different types of materials with the purpose to, e.g., overcome solubility problems of poorly water-soluble drugs, the pharmacokinetic and pharmacodynamic profiles of known drugs but also of new biomolecules, to modify the release profile of loaded compounds, or to decrease the risk of toxicity by providing site-specific delivery reducing the systemic distribution and thus adverse side effects. To succeed with the development of a nanopharmaceutical formulation, it is first necessary to analyze the type of drug which is to be encapsulated, select the type matrix to load it (e.g., polymers, lipids, polysaccharides, proteins, metals), followed by the production procedure. Together these elements have to be compatible with the administration route. To be launched onto the market, the selected production method has to be scaled-up, and quality assurance implemented for the product to reach clinical trials, during which in vivo performance is evaluated. Regulatory issues concerning nanopharmaceutics still require expertise for harmonizing legislation and a clear understanding of clinically compliant production methods. The first part of this study addressing “Nanopharmaceutics: Part I—Clinical trials legislation and Good Manufacturing Practices (GMP) of nanotherapeutics in the EU” has been published in Pharmaceutics. This second part complements the study with the discussion about the production scales and clinically compliant production methods of nanopharmaceutics.

## 1. Introduction

**Figure 1:**
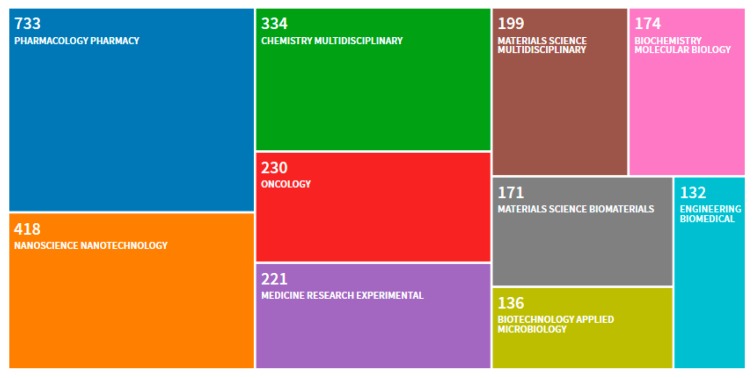
Distribution per scientific category of 2332 scientific works published between 2000 and 2020 using “clinical trials” and “nanoparticles” as keywords. 1274 research articles, 1001 reviews, 88 book chapters, 53 proceedings, 22 early accesses, 20 editorials, 11 meeting abstracts, 1 corrigendum. A total of 2341 publications are indexed in the Web of Knowledge (search on the 20 January 2020).

A number of emerging nanotechnologies are being exploited in medicine to improve the therapeutic outcome of several drugs and biomolecules and to address unmet medical needs. European Commission aims to lead innovation towards the development of these nanopharmaceutics by launching several funding opportunities within Member States, Associated Countries and Third Countries. The strategic plan for the next Horizon Europe framework programme has clearly set nanomedicines and advanced therapies as priorities. To succeed, the nanoproduct needs to be manufacturable at large scale and its quality assured in order to reach clinical trials. The topic is indeed of high scientific interest considering the number of scientific papers dealing with clinical trials and nanoparticles over the last twenty years (Figure 1).

The scientific category “Pharmacology Pharmacy” clearly dominates with 31.432% followed by the “Nanoscience Nanotechnology” with 17.925%. Regulating nanopharmaceutics is a challenge since the selection of the regulatory pathway is governed by the classification of the product which is defined by its type of action [1]. If the nanopharmaceutical product is defined as a product for advanced therapy, the procedure of marketing introduction authorization shall be submitted through a centralized procedure [2,3]. This implies harmonization and, thus, confidence in the quality and safety of the products. 

**Figure 2:**
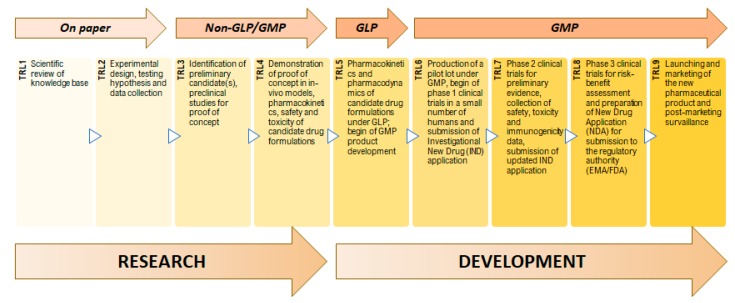
Technology Readiness Levels (TRL) and steps of research and development of a pharmaceutical product.

To measure the maturity of the development of a product, Technology Readiness Levels (TRL) can be used (Figure 2). This system estimates the stage of technical development based on quality parameters and is ranked in nine levels. TRL1 and TRL2 are related to basic research and to a very low level of experimental work. According to the needs and if studies of analytical and laboratorial nature are employed with a parallel settling of a model of proof-of-concept, the TRL3 is achieved. When the demonstration of efficacy of the process is set in vivo, the process is defined as optimized and achieves TRL4. The next transition occurs after implementation of Good Manufacturing Practices (GMP) which is realized by strict and precise tests undergone under a similar-to-reality milieu since there is a high probability of the nanopharmaceutical to get into the clinical trials phase (TRL5). TRL6 comprises the production of a batch according to GMP requirements to be available for Phase 1 of clinical trials [4]. In this case, an evaluation of some parameters as pharmacokinetics and pharmacodynamics is performed and the nanopharmaceutics are defined as a drug system model, being given the second proof-of-concept. Once entered the clinical trial phase, the model cannot be modified. Regarding TRL7, a scale-up process is needed to be structured according to GMP to be accepted in a Phase 2 of clinical trials related to safety evaluation [5,6]. TRL8 refers to Phase 3 of clinical trials and market introduction authorization, and TRL9 refers to actions after the approval. The product is officially on the market [7,8]. 

## 2. Production Scales of Clinically Compliant Nanopharmaceutics

The number of nanopharmaceutics currently on the market is still limited. This is mainly due to difficulties encountered during the processes of scaling-up which reflects on the quantity and even quality of products reaching clinical trials. The transition between the laboratory experimental production to the industry large-scale production is still a challenge in nanopharmaceutics. 

The laboratory-scale batches produced at the early stage of the developmental process are of very small size (usually 100–1000-times less than the industrial scale), and commonly result from pre-formulation studies, help to define the qualitative and quantitative formulation, and set the production parameters for medium/large scale. Such small batches supply the pre-clinical and/or clinical studies. The pilot scale batches are larger than the laboratory batches and usually support stability studies, and help to optimize production parameters and appropriate equipment. Pilot batches may also supply clinical trials. Industrial scale batches are those produced over the course of the marketing process. The scale-up of nanopharmaceutics may be a little more tricky as the process may affect the properties of the particles which make them singular in comparison to their bulk counterparts, e.g., colloidal stability, the drug loading, the mean particle size, the morphology, and surface properties [9]. The control of these properties is instrumental to ensure that the industrial batch will have the same physicochemical, pharmacokinetic and biopharmaceutical properties as the laboratory-scale batch. As these properties are strongly dependent on the production process, any deviation is only noted when the volume of batches is amplified. Good Manufacturing Practices (GMP) must be ensured over the course of the scaling-up [10] and the production process optimized to limit substantial differences between batches. The selection of the production process is governed by the type of nanomaterial, which is then dependent on the drug to be loaded and on the administration route. Besides, the low toxicological risk of the product must also be ensured before it gets into clinical trials [11].

## 3. Production Methods of Clinically Compliant Nanopharmaceutics

**Figure 3:**
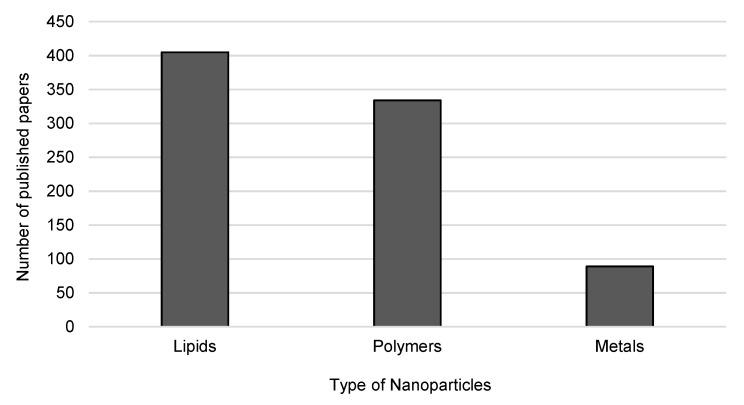
Publication trends on the types of nanoparticles being developed for clinical trials from 2000 to 2020. Source: Web of Knowledge, keywords: “lipid nanoparticles” or “polymeric nanoparticles” or “metal nanoparticles” and “clinical trials” (search on 20 January 2020).

The production methods of nanopharmaceutics should ensure that the product has at least one dimension in the nanoscale—from 1 nanometer to 100 nanometers—to conform with the definition [9,12]. Regarding the breakthrough that nanomaterials represent to pharmaceutical industry, the investment in novel approaches to develop improved pharmaceuticals is considered of high value. The main types of nanoparticles with potential to reach clinical trials are those composed of polymers, lipids and metals. Figure 3 shows the publication trends on these types of nanoparticles over the last twenty years.

Lipid nanoparticles can be of different types (e.g., liposomes [13,14,15,16], nanoemulsions [14,17,18], solid lipid nanoparticles (SLN) and nanostructured lipid carriers (NLC) [19,20,21,22,23,24]), each produced from very different lipids (e.g., phospholipids, synthetic oils, essential oils from plants, fatty acids, di-, mono-, and triglycerides, cholesterol), commonly resembling those existing in the human body and also in food. Due to their lipid composition, these particles are usually referred to as biocompatible, biodegradable and are generally recognized as safe [25,26,27]. These particles are specifically tailored to load lipophilic drugs [28], but the number of examples of hydrophilic including peptides and proteins [29,30], and amphiphilic compounds loaded in lipid nanoparticles is impressive. SLN and NLC receive special attention as, due to their solid matrix, they usually show modified release profile [31,32,33,34], and can be surface-tailored for site-specific targeted delivery [35,36]. 

Polymeric nanoparticles can be obtained from natural, semi-synthetic or synthetic polymers (e.g., chitosan [37,38,39], polylactic acid (PLA), poly(lactic-co-glycolic acid) (PLGA) [40,41,42,43]), which will then govern the way the drugs are encapsulated inside the matrix (dissolved or dispersed) or attached onto the nanoparticle’ surface (chemically bound or adsorbed), and how the drug is released [44]. Polymeric nanoparticles can be produced with a variety of sizes and shapes, with high drug payload for both hydrophilic and lipophilic molecules, can be surface-modified to increase the plasma half-life (e.g., PEGylation [45,46]) to have site-specific targeted delivery [47], and release the drug in a controlled fashion [48,49,50]. 

Metal nanoparticles are commonly employed in medical imaging and diagnostics, but also as a theragnostic approach (i.e., combination of therapy and diagnosis). Besides, some metal nanoparticles exhibit antimicrobial activity being commonly applied in coatings for wound treatment [51,52]. 

**Figure 4:**
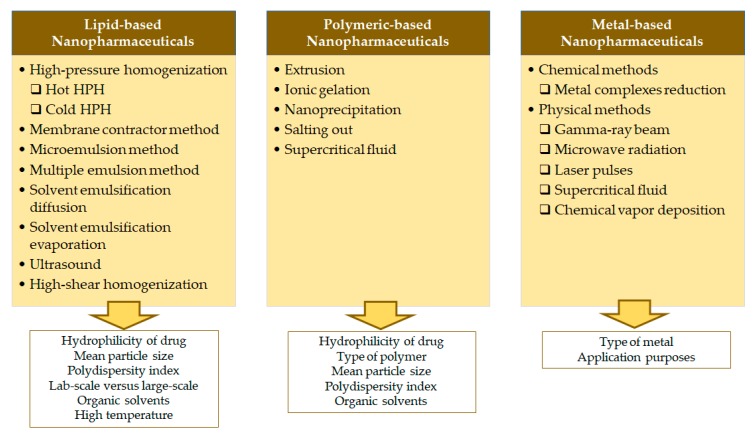
Most commonly used methods for the production lipid-, polymeric-, and metal-based nanopharmaceutics, and the critical factors determining their choice.

This section details the most commonly used methods for the production of each type of nanoparticles illustrated in Figure 4.

### 3.1. Lipid-Based Nanopharmaceutics

#### 3.1.1. High-Pressure Homogenization

High-pressure homogenization (HPH) is a technique with recognized advantage for large-scale production of lipid nanoparticles. Hot homogenization or cold homogenization can be used [53]. In the hot homogenization process, first the lipid is melted (in which the drug is dissolved or dispersed) and then, under mechanical stirring, is added to an aqueous surfactant solution at identical temperature [54]. The obtained emulsion is poured into the high-pressure homogenizer at a certain pressure (usually 500–600 bar) for some minutes (c. 3–5 min) and homogenized at high temperature (usually 5–10 °C above the melting point of the solid lipid). The resulting oil/water (O/A) nanoemulsion is cooled down to room temperature in order to crystallize the liquid lipid to solid lipid and generate the lipid nanoparticles [55,56,57,58]. For thermo-sensitive or hydrophilic drugs, the cold homogenization process is usually recommended. In this approach, a first step to melt the lipid is also needed in order to disperse the drug followed the fast cooling of the mixture. The obtained solid mixture is then ground in a mortar mill to obtain lipid particles. These are dispersed in an aqueous surfactant solution at room or lower temperature to prepare a suspension, and then processed in the high-pressure homogenization using the same processing conditions as mentioned above but at room temperature. The cold process usually originates lipid nanoparticle dispersions with higher polydispersity than the hot process [33,59].

#### 3.1.2. Membrane Contractor Method

The membrane contractor method also requires a first step of melting of the solid lipid in which the drug is dispersed or dissolved [60]. This organic phase is mixed by mechanical stirring in an aqueous surfactant solution to obtain a hot emulsion which is then pressed against the membrane applying the required pressure so that the inner oily droplets are sized down when crossing the membrane, forming very small droplets which recrystallize when in contact with a cold aqueous phase. This method generates monodispersed nanoparticles and can be scaled-up with some adaptations.

#### 3.1.3. Microemulsion Method

The microemulsion method requires the preparation of a microemulsion by dispersing, under mechanical stirring, the melted lipid containing the drug in an aqueous surfactant solution heated up at the same temperature as the organic phase, followed by the dilution in a large volume of cold water (0–4 °C) under magnetic stirring. Lipid nanoparticles are result from the recrystallization of the lipid phase induced by the thermal shock. Although not particularly suited for large-scale production, the microemulsion method is simple, reproducible and suited for sensitive compounds [61]. To produce a stable microemulsion, a co-surfactant added to the inner lipid phase is usually needed. Some new adaptations, e.g., replacement of first heating step by microwave treatment to disperse the lipid in the aqueous phase, have been proposed [62].

#### 3.1.4. Multiple Emulsion Method

Multiple emulsion or double method has been proposed for the loading of hydrophilic molecules into lipid matrices [63]. It requires the preparation of a water-in-oil (w/o) emulsion by dispersing the aqueous inner phase containing the drug into the organic phase obtained from the dissolution of the solid lipid in a suitable organic solvent, followed by the dispersion of this w/o emulsion into an aqueous surfactant solution to produce a water-in-oil-in-water (w/o/w) emulsion. By evaporation of the organic solvent under gentle mechanical stirring lipid nanoparticles are generated. This method is also not particularly suited for the production of large volumes of particles (with the additional limitation of usage of organic solvents), but it is reproducible and can be an interesting option for the production of small batches to feed pre-clinical and clinical studies. 

#### 3.1.5. Solvent Emulsification Diffusion

The solvent emulsification diffusion method has been firstly proposed for the production of polymeric nanoparticles [64], and then adapted to produce lipid nanoparticles. Briefly, it is based on the dispersion of an organic solution of the lipid in a polar protic or aprotic organic solvent (e.g., ethanol, acetone) in an aqueous surfactant solution. The diffusion of the organic solvent from the inner phase in contact with the water phase, under gentle stirring results in the formation of lipid nanoparticles. This approach is limited to small-sized batches, but it has the advantage of not requiring heat, and thus is interesting for sensitive compounds.

#### 3.1.6. Solvent Emulsification Evaporation

This method is a variation of the solvent emulsification diffusion by replacing the polar protic or aprotic organic solvent by non-polar solvent (e.g., chloroform, dichloromethane). It is suited for the loading of lipophilic drugs into the lipid nanoparticles but with higher risk of toxicity due to the type of solvents involved.

#### 3.1.7. Ultrasonication and High-Shear Homogenization

These methods commonly precede the high-pressure homogenization technique as they are used for the production of the pre-emulsion prior to high-pressure homogenization. If used isolated, they usually need extended times for the dispersion of the melted lipid phase into the hot aqueous surfactant solution for the production of the pre-emulsion, which upon cooling down generates lipid nanoparticles with a higher polydispersity, in comparison to the combination of ultrasound/high-shear homogenization with the high-pressure homogenization [65,66,67].

### 3.2. Polymeric-Based Nanopharmaceutics

#### 3.2.1. Extrusion

The production of polymeric nanoparticles by extrusion method requires the use of polycarbonate membranes and is based on the induced precipitation of drug-loaded nanoparticles at the exit of the nanopores [68]. The method ensures a high reproducibility.

#### 3.2.2. Ionic Gelation

Ionic gelation is commonly used in the production of nanoparticles from ionic polymers (e.g., chitosan and tripolyphosphate, alginate and dextran sulphate). Briefly, the method involves two mixed aqueous phases with a following transition from a liquid to a gel—a consequence of ionic interactions occurring at room temperature [69]. The generated nanoparticles usually have sizes below 500 nm being nevertheless governed by the type of polysaccharides used as particle matrix. This method also ensures high reproducibility.

#### 3.2.3. Nanoprecipitation

The nanoprecipitation technique is a bottom-up method which generates nanoparticles instantaneously using two miscible solvents, one of which does not dissolve the polymer. Firstly, the drug and the polymer are dissolved in one of the solvents (i.e., the one dissolving the polymer). Nanoprecipitation of nanoparticles loaded with drug happen under gentle magnetic stirring by dropwise addition of the solvent in which the polymer is not soluble. This latter solvent is called non-solvent [70].

#### 3.2.4. Salting-Out

The salting-out method is a variation of the nanoprecipitation method by replacing the non-solvent by an agent that induces the precipitation of the polymer entrapping the drug. Briefly, the organic solvent in which the polymer and drug are dissolved (usually acetone or ethanol) is added to an aqueous surfactant solution containing a high concentration of electrolyte as the salting-out agent (usually, magnesium chloride, calcium chloride, magnesium acetate) to produce an aqueous gel forming oil-in-water emulsion under high mechanical stirring [67]. The dilution of this emulsion in a volume of water appropriate for the diffusion of solvent into aqueous phase, decreasing the ionic strength in the electrolyte. The diffusion of the organic solvent induces the hardening (or nanoprecipitation) of the polymer which entraps the drug and generates nanoparticles. The organic solvent can further be removed by reduced pressure. In a nonelectrolyte system, sucrose can be used as salting agent.

#### 3.2.5. Supercritical Fluid

The supercritical fluid method is based on the extraction of the organic solvent from the inner phase of an oil-in-water (o/w) emulsion using the supercritical carbon dioxide (CO_2_) [71]. This method has been reported to produce monodispersed nanoparticles with less residual organic solvent, and with high drug payload. The final material is reported to be a dried powder that facilitates the production of improved liquid or solid drug formulations, while the technique is described as environmentally friendly and with potential to be scaled-up. This method is also being adapted for the production of lipid nanoparticles [72].

### 3.3. Metal-Based Nanopharmaceuticals

Metal-based nanoparticles are produced from bottom-up techniques [9,73], either from chemical or from physical methods. In the chemical methods, the reduction of metal complexes in diluted solutions is preferred, whereas in physical methods a vast array of techniques has already been employed, e.g., gamma-ray beam, microwave radiation, laser pulses, supercritical fluids and deposition by chemical vapor [74].

## 4. Requirements for Clinically Accepted Nanopharmaceutical Batches

The scaling-up of a production process requires an absolute control or each and every technical parameter, in such a fashion that only slight differences can be found between different batches of the same nanoproduct. To assist on the development of clinically accepted nanopharmaceutical batches, the “*guideline on the requirements to the chemical and pharmaceutical quality documentation concerning investigational medicinal products in clinical trials*”, of the Eudralex, Volume X, is of instrumental value as it displays specifications about the development of IMP (Investigational Medicinal Products) [75]. Examples of currently ongoing or upcoming clinical trials involving the use of nanopharmaceuticals are listed in Table 1.

The nanoproduct to reach clinical trials needs to be carefully identified regarding the Annex 13 of IMP. Besides, the number of individuals enrolled in the clinical trial must be critically defined so that the tested batch size is aligned with the respective phase of the trial. In Phase I of clinical trials, the group size is usually between 10 and 100 [76]. Phases II and III require higher number of subjects to determine the parameters of safety and control of produced batches to prepare the Investigational Medical Product Dossier (IMPD).

Once the clinical trials are finished, the results are included in the IMPD to further submission for marketing introduction authorization [77]. In Europe, the Common Technical Documentation has a comparable format as the IMPD [78,79].

## 5. From Nanopharmaceutics to Nanonutraceutics: A Bet for the Future

Over the last decades, technological developments gave birth to a new class of products, the so-called nanopharmaceutics. These formulations represent a step forward to innovative personalized medicines with improved outcomes to patients and to public health systems. However, with innovation and modernization, come also other issues related to regulatory affairs requiring new legislation to shape them for human use, which should cover quality, efficacy and safety of the product before it reaches clinical trials. A positive benefit/risk relationship must be ensured.

**Figure 5:**
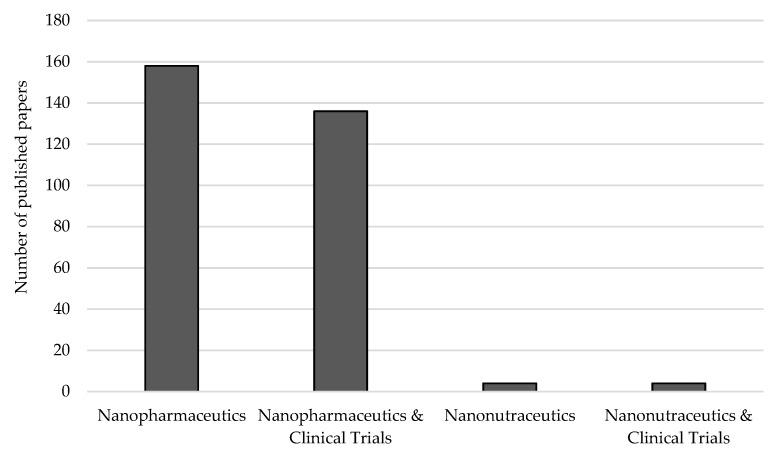
Number of papers indexed in the Web of Knowledge dealing with nanopharmaceuticals, nanonutraceuticals and clinical trials. Source: Web of Knowledge, keywords: “nanopharmaceuticals or nanopharmaceutics” and “nanonutraceuticals or nanonutraceutics” and “clinical trials” (search on 20 January 2020).

The internationally available guidelines for clinical trials and the required IMPD are instrumental to ensure that the product submitted to an evaluation for a marketing introduction authorization is reliable. While the production of nanopharmaceutics are strongly tied to GMP, continuous scientific guidance is still required to ensure quality and safety. As the methods for production of nanopharmaceutics differ amongst the advantages and easiness to be scaled-up, the selection should also rely on the safety of the final product. Bioactives from natural sources with nutraceutical value (of vegetal or animal food matrices) are of focused interest and are being proposed as ingredients to be loaded into nanoparticles to obtain a new production—nanonutraceutics. The added value of several nutraceuticals in the prevention, treatment or delay the onset of a disease is very well documented [80,81,82,83,84], although much research is still needed as seen by the number of publications indexed in the Web of Knowledge dealing with nanonutraceutics and clinical trials (Figure 5).

Nutraceutics, which derive from food matrices of vegetal or animal matrices, are a novel toolbox not yet completely explored for its full potential in medicine. Current research is looking towards several nanotechnological approaches to be exploited for the formulation of nutraceuticals [84,85,86,87,88,89,90,91,92,93], and to build up the emerging area of the nanonutraceutics [9,94,95,96]. Nutraceutics, a portmanteau of the words ‘nutrition’ and ‘pharmaceutical’, have been recently defined as “the phytocomplex if they derive from a food of vegetal origin, and as the pool of the secondary metabolites if they derive from a food of animal origin, concentrated and administered in the more suitable pharmaceutical form” [84,97]. Nanonutraceutics could be an important tool useful among the strategies adopted in managing health conditions, particularly tailored to patients who are not eligible for a conventional pharmacological therapy. Studies on follow up, use, and compliance of pharmaceuticals reported in the area [98,99,100,101], as well as communication strategies and assessment [102], should be extended also to nutraceuticals and carried out in view of exploiting the field to different health conditions, e.g., the ones clustered in the so-called “metabolic syndrome”, which includes conditions ranging from obesity to dysmetabolism [103,104,105]. These latter are often related to the food intake/dietary habits of each person. The efficient encapsulation of nutraceuticals, their smart delivery and release from a nanoformulation are the emerging challenge of nanotechnology applied to food derived products. To address this issue, the principles of nanotechnology should be used for the proficient delivery of nutraceuticals with the objective to improve their bioavailability thereby increasing health benefits. To reach this end point, extensive research on encapsulation of nutraceuticals into biodegradable, environment friendly nanocarriers, is ongoing to increase their absorption and the therapeutic potential. This aspect is challenging and attracting growing interest for its perspective potential, even if further studies are needed to assess whether to a nano-level changes in physical and biochemical properties may occur. Nanonutraceutic products are a bet for the future. They should be assessed completely for retaining their nutraceutical properties at a nano level, guarantee safety and the maintenance of the GMP in the production processes, substantiating with scientific data their quality and stability, guarantee their safety and efficacy. Follow-up studies to evaluate possible unwanted effect, as it is needed also for both the nanopharmaceutics and nanonutraceutics [9,106,107,108].

## 6. Conclusions

Nanopharmaceutics emerged as a promising technology in pharmaceutical industry due to their unique properties resulting from the size, shape, morphology and surface properties, which are effective only if essential parameters such as quality, safety and efficacy are ensured over the course of the scale-up process. However, no standard methodology is available to control the quality of these nanoproducts in a scale-up process. Nanopharmaceutics triggered an entire revolution in pharmaceutical industry with significant impact also on nutraceutics, which are attracting growing interest for their beneficial health effects, resulting from improved delivery, enhanced bioavailability and biological effect. Although several tremendous investments from industrial stakeholders have already been made, future outcomes will positively accompany the modifications in the way these products are controlled, produced, and launched on the market, through safe and effective filtering out of the non-compliant products and preventing them going to the market, and facilitating the good products being made available to the public, as well as stimulating the developments of even newer, and improved products.

## Figures and Tables

**Table 1:** Examples of currently ongoing or upcoming clinical trials of treatments containing nanopharmaceuticals (source: https://clinicaltrials.gov/).

Identifier	Title	Nanopharmaceutics	Purpose
NCT03752424	Topical Silver Nanoparticles for Microbial Activity	Silver nanoparticles Topical approved anti-microbial gel	Foot Infection Fungal Infection, Bacterial
NCT04000386	A Study of Efficacy of Zinc Oxide Nanoparticles Coated Socks in Prevention of Unpleasant Foot Odor	Zinc oxide nanoparticles coated socks Placebo socks	Zinc Oxide Foot Dermatoses
NCT03635138	Effect of the Incorporation of Copper and Zinc Nanoparticles into Dental Adhesives	Metal nanoparticles (Zn and Cu) Dental Adhesive pure	Caries, Dental
NCT03774680	Targeted Polymeric Nanoparticles Loaded with Cetuximab and Decorated with Somatostatin Analogue to Colon Cancer	Cetuximab nanoparticles Oral approved anticancer drug	Colon Cancer Colo-rectal Cancer
NCT03478150	Evaluation of the Antibacterial Effect of Laser Diode and Zinc Oxide Nano-Particles in Cavity Disinfection	Zinc oxide nanoparticles Laser diode	Caries, Dental
NCT03659864	The Role of Eicosanoids in the Cardiovascular Actions of Inhaled Nanoparticles	Diesel exhaust particulate Carbon nanoparticles Small graphene oxide	Blood Biomarkers Vasodilation Blood Clotting
NCT03666195	The Anti-microbial Effect of Titanium Dioxide Nano-Particles in Complete Dentures Made for Edentulous Patients	Titanium dioxide nanoparticles	Candida Infection Denture Stomatitis
NCT03550001	Carbon Nanoparticles (CNP) as Lymph Node Tracer in Rectal Cancer After Neoadjuvant Radiochemotherapy	Procedure: Injection CNP before NAT	Rectal cancer
NCT03003546	Nab-paclitaxel/Rituximab-coated Nanoparticle AR160 in Treating Patients with Relapsed or Refractory B-Cell Non-Hodgkin Lymphoma	Laboratory Biomarker Analysis Nab-paclitaxel/Rituximab-coated Nanoparticle AR160	Aggressive Non-Hodgkin Lymphoma CD20 Positive Recurrent B-Cell Non-Hodgkin Lymphoma
NCT04094077	Evaluating AGuIX^®^ Nanoparticles in Combination with Stereotactic Radiation for Brain Metastases	Brain Metastases	Drug: AGuIX
NCT03700489	Mycological Comparative Study on Maxillary Dentures of Two Different Materials	Effect of Tio2 Nanoparticles on Candida Aggregation	Other: titanium dioxide denture
NCT04138342	Topical Fluorescent Nanoparticles Conjugated Somatostatin Analogue for Suppression and Bioimaging Breast Cancer	Quantum dots coated with veldoreotide Topical approved placebo	Breast Cancer Skin Cancer Skin Diseases
NCT04148833	Treatment of Patients with Atherosclerotic Disease with Paclitaxel-associated to low-density lipoprotein (LDL)-Like Nanoparticles	Drug: LDE-Paclitaxel Drug: LDE-Placebo	Coronary Artery Disease Atherosclerosis Inflammation
NCT04240639	An Extension Study MRI/US Fusion Imaging and Biopsy in Combination with Nanoparticle Directed Focal Therapy for Ablation of Prostate Tissue	AuroShell particle infusion	Neoplasms of the Prostate
NCT03692286	Assessment of Postoperative Pain After Using Various Intracanal Medication in Patients with Necrotic Pulp	Silver nanoparticle/Calcium hydroxide Silver Nanoparticles in gel form Calcium Hydroxide Intracanal medication	Postoperative Pain
